# Characterization of structures in biofilms formed by a *Pseudomonas fluorescens *isolated from soil

**DOI:** 10.1186/1471-2180-9-103

**Published:** 2009-05-21

**Authors:** Marc M Baum, Aleksandra Kainović, Teresa O'Keeffe, Ragini Pandita, Kent McDonald, Siva Wu, Paul Webster

**Affiliations:** 1 Department of Chemistry, Oak Crest Institute of Science, 2275 E. Foothill Blvd, Pasadena, CA 91107, USA; 2 Electron Microscope Lab, 26 Giannini Hall, University of California, Berkeley, Berkeley, CA 94720, USA; 3 Ahmanson Advanced EM & Imaging Center, House Ear Research Institute, 2100 W. 3rd Street, Los Angeles, CA 90057, USA

## Abstract

**Background:**

Microbial biofilms represent an incompletely understood, but fundamental mode of bacterial growth. These sessile communities typically consist of stratified, morphologically-distinct layers of extracellular material, where numerous metabolic processes occur simultaneously in close proximity. Limited reports on environmental isolates have revealed highly ordered, three-dimensional organization of the extracellular matrix, which may hold important implications for biofilm physiology *in vivo*.

**Results:**

A *Pseudomonas *spp. isolated from a natural soil environment produced flocculent, nonmucoidal biofilms *in vitro *with unique structural features. These mature biofilms were made up of numerous viable bacteria, even after extended culture, and contained up to 50% of proteins and accumulated 3% (by dry weight) calcium, suggesting an important role for the divalent metal in biofilm formation. Ultrastructurally, the mature biofilms contained structural motifs consisting of dense, fibrillary clusters, nanofibers, and ordered, honeycomb-like chambers enveloped in thin sheets.

**Conclusion:**

Mature biofilms contained living bacteria and were structurally, chemically, and physiologically heterogeneous. The principal architectural elements observed by electron microscopy may represent useful morphological clues for identifying bacterial biofilms *in vivo*. The complexity and reproducibility of the structural motifs observed in bacterial biofilms appear to be the result of organized assembly, suggesting that this environmental isolate may possess ecological advantages in its natural habitat.

## Background

Bacteria possess the ability to adhere to surfaces and grow within an extracellular matrix of their own synthesis. Although these bacterial aggregates, or biofilms, were first identified in natural aquatic environments [1], their importance in infectious disease is attracting much attention [2-4]. For pathogens, life in a biofilm offers protection from mucociliary clearance, phagocytosis, and from antibiotic attack [3,5,6], thereby playing a participatory role in persistent infections [2].

Bacteria are thought to organize into communities that produce and populate the biofilm, controlling its morphology by varying growth and gene expression, and by interacting with neighboring cells. Random environmental pressures also participate in shaping these specialized structures [7]. Chemotaxis and bacterially induced small-scale water currents [8,9] have been used to explain large (0.3–0.5 mm in diameter) periodic bacterial patterns on mucus veils suspended over sulfidic marine sediments [10]. Surface-bound biofilms have been observed to develop into microscopic structures, such as the pillars and mushroom-shaped cell clusters produced by *Pseudomonas aeruginosa *[11]. *Pseudomonas fluorescens *SBW25 produced biofilms that were comprised of extensive, extracellular non-periodic webs of fine (< 20 nm wide) cellulose fibers [12]. Freeze-dried colonies of *Erwinia amylovora *were found to contain cross-linked stalactites of extracellular polymeric substances (EPS) with an approximate spacing of 10 μm [13], and biofilms of *Listeria monocytogenes *strains consisted of complex, regular structures with an approximate spacing of 50 μm [14].

The organism studied in the present report is a *Pseudomonas fluorescens *soil isolate from an environment heavily contaminated by tar seeps. *P. fluorescens *is a ubiquitous, Gram-negative, motile, biofilm-forming bacterium commonly-encountered in soil and water habitats. The organism plays an important role in food spoilage, drinking water quality, plant disease, and nosocomial infections. *P. fluorescens *also is known to form biofilms and consequently the surface adhesion of a number of isolates has been investigated. Cossard *et al*. determined that the adherence properties of four *P. fluorescens *isolates were independent of their ecological habitat [15]. *P. fluorescens *WCS365 was found to produce a cell surface protein (LapA) that promoted the colonization of glass, plastic, and quartz sand *via *adhesion [16]. Biofilm formation by *P. fluorescens *SBW25 at the air-liquid interface required an acetylated form of cellulose [12] and the genetic systems that underpin cellulose production and colonization in numerous strains have been determined [17,18]. The physiology and behavior of *P. fluorescens *biofilms under diverse hydrodynamic stresses have been the subject of numerous flow-chamber studies [19-22]. Biofilms formed under a turbulent flow regime were more active and contained more viable biomass than their laminar counterparts. Given *P. fluorescens*' resistance to a number of bacterial agents, biofilm control methods involving bacteriophages have been investigated recently with encouraging preliminary results [23]. Studies on biofilms produced by *P. fluorescens *have relied heavily on optical microscopy, notably on selective staining with fluorescent dyes followed by examination with confocal laser scanning microscopy. Plasmid expression of specially-constructed autofluorescent proteins also has been used to image *P. fluorescens *strains in the rhizosphere [24,25] and on leaf surfaces [25,26].

Recent studies on biofilms formed by a pathogenic strain of *Staphylococcus epidermidis *have revealed highly ordered, three-dimensional organization of extracellular matrix that was vacated as the biofilm matured [27]. If the remarkable ability to form complex extracellular structures were restricted to one strain of pathogenic bacteria, it would constitute an interesting observation with limited applicability. Here we demonstrate that a strain of bacteria isolated from a natural environment can produce biofilms consisting of complex, organized structures.

## Results

### The bacterial isolate is an axenic Pseudomonad

The environmental isolate used in this study, EvS4-B1, consisted of Gram-negative, rod-shaped (0.5 × 1.4 μm in stationary phase) cells that produced fluorescent colonies on Gould's S1 agar. To ensure that axenic cultures were examined, the bacterial populations were propagated and PCR was performed using a universal primer that amplifies a consensus 16S rRNA gene, and a primer that identifies a *Pseudomonas*-specific amplicon within the 16S rRNA gene. The 16S rRNA gene sequence of EvS4-B1 was found to be 99% identical (1248/1249, for the general primer; 881/882 for the *Pseudomonas*-specific primer) to the corresponding region of *P*. sp. TM7_1. Metabolic tests and fatty acid analysis identified EvS4-B1 as belonging to the *P. fluorescens *species (metabolic: *% ID*, 99.7; *T*, 0.87; FAME: *SI*, 0.642). The culture was free from contaminating species based on the purity of the above sequence.

### Bacteria growing *in vitro *form biofilms with reproducible macroscopic features

Initially, axenic cultures of the bacterial isolate propagated exponentially, but the optical density of the growth medium started to decline significantly 24 h following inoculation [see Additional file 1]. The drop in planktonic bacterial numbers, estimated by optical density, coincided with the formation of macroscopic opaque structures in the bottom of the culture tube. These structures had a diaphanous, gossamer appearance [see Additional file 2] and consisted of a dense, fibrillary core, with interdispersed white flocs that usually were anchored firmly to the bottom of the tube when grown as standing cultures; in shaking cultures, the material was commonly detached from the bottom of the tube. It was concluded that the structures in the bottom of the tubes were biofilms.

**Figure 1:**
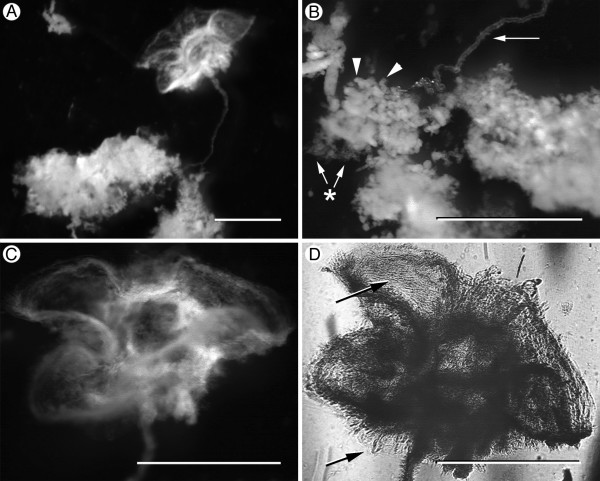
***P. fluorescens *EvS4-B1 biofilms (21 days) contain macroscopic 3-dimensional structures**. (A) Gentle disruption of the biofilm revealed a fragile mass of amorphous material connected to a parachute-like structure. (B) The structures were either well-defined packets (arrowheads) or aggregated flocs (asterisk) anchored to a fibrillary core (arrow). (C) The parachute-like structure was made up of 5 or 6 compartments. (D) Backlighting highlighted the fibrous nature of the parachute-like structure (arrow). Scale bars = 1.5 mm.

Examination of the mature (between 1 and 3 weeks old) hydrated biofilms in a dissecting microscope revealed macroscopic features that were reproducible from culture to culture. An aggregation of delicate flocs of opaque material made up the bulk of the biofilm volume (Fig. 1A and 1B). Tethered to this construct *via *a thin cord was a parachute-like appendage approximately 2 mm in diameter (Fig. 1C) that consisted of material resembling fibrous sheets (Fig. 1D). While each culture only contained one of these highly unusual parachute-like structures, they were consistent macroscopic biofilm features when *P. fluorescens *EvS4-B1 was grown in minimal media. Glutaraldehyde fixation of the biofilms led to rapid dissolution of the flocculent material and slowly dissolved the fibrous, string-like core. The parachute-like appendage was the only biofilm component that remained after aldehyde fixation and subsequent staining and dehydration.

### Biofilms formed by the bacterial isolate have a complex ultrastructural morphology

**Figure 2:**
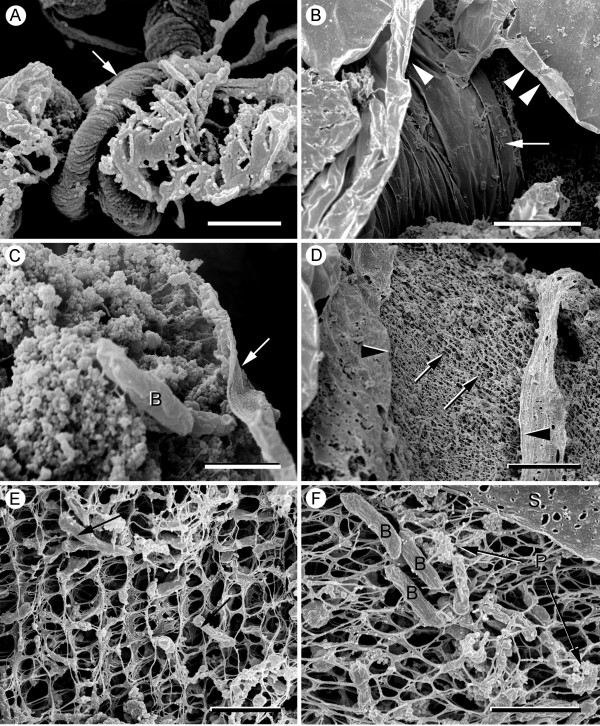
**Scanning electron micrographs of *P. fluorescens *EvS4-B1 biofilms (14 days) prepared using cryomethods**. (A). Fibrillary structures appeared to be made up of twisted fibers (arrow) scale bar = 1 μm. (B). Flat sheets of material (arrowhead) also were observed. Some of the sheets seemed to be wrapped around other structures (arrow); scale bar = 20 μm. (C) The inside core of the "wrapped" structures consisted of bacteria, [B], embedded in an extracellular matrix of particulate matter and a thin sheet of material (arrow); scale bar = 1 μm. (D) The outer sheet (arrowheads) enveloped an inner core consisting of fibers forming irregular network-like structures (arrow); scale bar = 10 μm. (E) The network consisted of fibers arranged in a periodic pattern. The bacteria (arrows) were two to three times larger than the spaces in the network; scale bar = 2 μm. (F) A sheet of material, [S], covered the fiber network and was attached to it. The fibers were associated with bacteria, [B], and particulate matter, [P]; scale bar = 2 μm.

*P. fluorescens *EvS4-B1 biofilms were prepared for SEM analysis using cryomethods. Conventional aqueous cross-linking and contrasting agents, such as glutaraldehyde and osmium tetroxide, were not used because of the structural disruption we observed under the dissection microscope as described above. Low magnification SEM examination of the prepared biofilms revealed unique structural features (Fig. 2). Running through the biofilm were cords of twisted material (Fig. 2A). Larger structures consisting of wrapped sheets also were present inside the biofilm (Fig. 2B). When specimen preparation led to breaks in this structure, the biofilm core was exposed (Fig. 2C) and consisted of small numbers of bacteria embedded in a matrix of fibers and particulate matter aggregating on the fibers (Fig. 2C). In other parts of the biofilm, the fibers were more apparent and formed irregular, net-like structures (Fig. 2D). At higher magnification it was possible to see that the fibers were organized into ordered networks of periodic nets. These nets contained few bacteria (Fig. 2E) and were covered by thin sheets of material similar to that observed around the bacteria embedded in the particulate matter (Fig. 2F).

### The ultrastructures observed by SEM are not artifacts resulting from sample preparation

**Figure 3:**
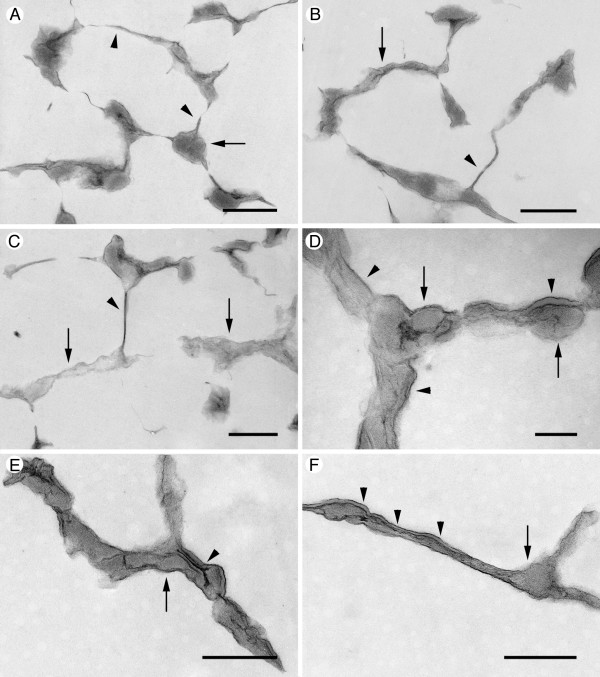
**Transmission electron microscopy images of *P. fluorescens *EvS4-B1 biofilms (21 days)**. Specimens were prepared using cryomethods and embedded in resin. The sections represent regions of biofilm containing structured networks of fibers and sheets, but few bacteria. (A) The walls consisted of thin laminar structures (arrowhead) with globular material (arrow) accumulating in branching regions; scale bar = 500 nm. (B) In other regions of the biofilm, the wall-like structures had different thicknesses. The thin walls (arrowhead) were attached to thicker walls (arrow); scale bar = 500 nm. (C) Different wall morphologies consisted of thin, straight walls (arrowhead) branching from thicker walled structures (arrows); scale bar = 500 nm. (D) The thicker walls were composed of globular amorphous masses (arrows) covered in part by a distinct coating (arrowheads); scale bar = 200 nm. (E) and (F) The different components of the thicker walls consisted of globular masses (arrows) separated by and covered with thin coatings (arrowheads); scale bar = 500 nm.

The transmission electron microscopy (TEM) images of the embedded biofilms (Fig. 3) are consistent with the corresponding SEM data (Fig. 2) and therefore validate the ultrastructural organization observed in the SEM suggesting that they did not result from sample preparation. The honeycomb-like structures, as well as the morphology of the partitions, are clearly visible using both techniques. The structures appeared to have two types of walls. Either it was thin with a smooth surface, or it was thicker and made up of globular structures (Fig. 3D–F). The thicker walls, although smooth on the surface, were of variable thickness giving them a bumpy appearance (Fig. 3D–F). The section staining revealed separations between the components of the thicker walls and globular masses separated by thin sheets (Fig. 3E–F). No obvious freezing damage due to ice crystal formation was observed suggesting that the EM data presented here are of real ultrastructural features in the biofilms and are not the result of eutectic crystallization.

### Biofilms are chemically heterogeneous

Hydrated biofilms from multiple cultures were combined taking care to minimize the inclusion of spent media without disturbing the fragile structures. No further handling of the biofilms was carried out prior to freeze-drying in order to preserve the chemical integrity of the structures. Physical or chemical treatments of the samples such as centrifugation, filtration, extraction, and ion exchange chromatography have the potential to significantly alter the biofilm composition, thus biasing the results of the chemical analysis. The method described here is simple, convenient, minimally invasive, and is designed to provide representative samples for compositional analysis. Hydrated biofilms (0.9189 g) afforded 15.6 mg of dry material (16.0 mg g^-1^) consisting of biofilm **and **spent media, where-as spent media free of biofilm (1.9255 g) afforded 10.8 mg of dry material (5.6 mg g^-1^). Assuming that the dry material makes up a negligible proportion (1.7% in the case of biofilm plus media) of the mass of the hydrated sample, the media contribution to the mixed sample was estimated as 5.2 mg (0.9189 × 5.6), or 33% [(5.2/15.6) × 100%]. Background contributions from spent media to the chemical sample make-up were subtracted from the mixed biofilm-media samples according to eq. 1. This simple relationship was employed throughout to estimate biofilm composition. Results of the biofilm chemical analyses are summarized in Table 1.

**Table 1:** Biofilm chemical composition.

Analyte	Analysis method	Mass concentration (μg mg^-1^)^a^
Calcium	ICP-AES	29.9
Magnesium	ICP-AES	10.1
Total proteins	UV absorption	490
Total proteins^b^	Folin reaction (Lowry assay)	240
Acidic polysaccharides^c^	Phenol-sulfuric acid reaction	79
Neutral polysaccharides^c^	Phenol-sulfuric acid reaction	67
Nucleic acids	UV absorption	46
DNA	DAPI-fluorescence	5.4

^a^Dry material.

^b^Measured as BSA.

^c^Measured as dextrose monohydrate.

The principal IR absorption bands of the mixed biofilm/media sample are presented elsewhere [see Additional file 1]. The most striking difference between the biofilm/media and media spectra is the presence of the peak at 1536 cm^-1 ^in the biofilm-containing sample, which is virtually absent in the media sample. This peak likely corresponds to an amide II stretch in proteins [28-30]. The biofilm-containing sample lacks peaks at 2814, 1930, 1359, 1200,1191, and 940 cm^-1^, which all are present in the media sample. The relative *β*-*D*-mannuronate (M) and *α*-*L*-guluronate (G) content of alginate copolymers can be estimated as the M/G ratio using the absorption bands at 1320 and 1290 cm^-1 ^[31]. The corresponding bands observed here were at 1315 and 1275 cm^-1 ^and were weak, suggesting a low alginate content. Strong absorptions in the 1064–1078 cm^-1 ^range assigned to vibrations in polysaccharide ring structures [28] also were missing. Although a very weak shoulder at 1745 cm^-1 ^was observed, neither the biofilm nor the media IR spectra exhibited significant peaks around 1728–1724 cm^-1^, which correspond to the C = O stretch in O-acetyl esters [28], specifically acylated sugars.

### Biofilms contain viable bacteria and glycoproteins

**Figure 4:**
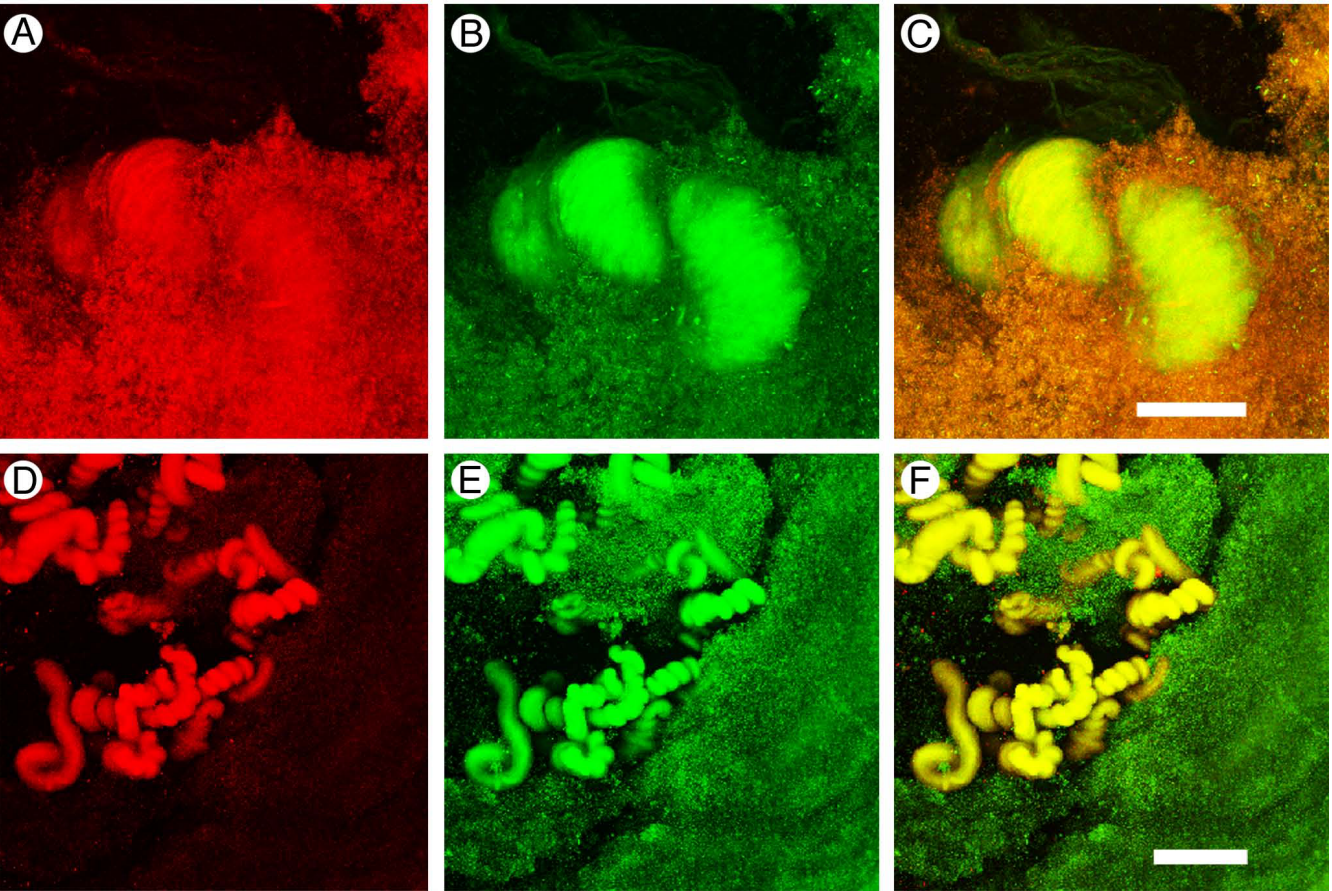
**Confocal images of *P. fluorescens *EvS4-B1 biofilms (7 days) labeled with the Live/Dead stain (A-C) and with concanavalin A/Syto 9 (D-F)**. (A) Propydium iodide labeled dead bacteria. (B) Syto 9 labeled live bacteria. (C) The two images merged; scale bar = 50 μm. (D) Concanavalin A labeled coiled structures (arrow). (E) Syto 9 labeled bacteria. (F) The two images merged; scale bar = 50 μm.

The primary goal of the confocal laser scanning microscopy (CLSM) studies was to determine if viable bacteria were present in the mature biofilm structures. CLSM in combination with multiple, chemo-specific, fluorescent labels are increasingly being used to achieve *in situ *characterization of bacterial biofilms with up to single cell resolution [32-34]. Biofilms from *P. fluorescens *EvS4-B1 cultures were labeled with BacLight and were examined by CLSM. This technique optimizes the possibility of detecting intact, viable bacteria that may be un-culturable on agar plates or as planktonic forms in liquid medium. The labeling demonstrated that the bacterial biofilms contained significant populations of living bacteria in clusters surrounded by dead bacteria (Fig. 4A–C). These results indicate that the mature biofilms are still physiologically active and are not merely aggregates of cellular debris.

Concanavalin A (Con A) is one of the most widely used and best characterized lectins in biomedical research. It has a broad applicability because it binds to alpha-linked mannose residues, a common component of the core oligosaccharide of many glycoproteins. The presence of Con A binding is usually an indication that glycoproteins are present. Con A binding was observed in many regions of the biofilm that also contained bacteria, as determined by Syto 9 staining (Fig. 4D–F). The cords of twisted material running through the biofilm were easily recognizable features of the biofilm that labeled with Con A (Fig. 4D–F). These cords appeared to be embedded in aggregates of bacteria that did not label with Con A. The structures that labeled with Con A in other regions of the biofilm appeared diffuse and were not easily identified (data not shown).

## Discussion

A bacterial species from an extreme environment rich in toxic compounds was isolated into axenic culture and grown in the laboratory. During the course of these studies, it was observed that the isolate produced atypical growth curves and formed a macroscopic structure tethered to the bottom of the culture tubes. These biofilms were unusual as they did not consist of the typical mucoidal material, but were made up of well-defined solid structures. Confocal laser scanning microscopy confirmed that these mature structures contained significant zones of physiological activity. Physical and chemical characterization of the mature biofilms was carried out and is discussed below.

When examined by light microscopy, bacterial cultures reproducibly contained similar structural motifs that were composed of viable bacteria as well as dead cells and extracellular material. At the macroscopic level, delicate flocculent material of what appeared to be bacterial aggregates was enveloped by a network of fibers. Smaller fibers branched from this central core in a microscopic analogue to tree branches emanating from a trunk and surrounded by foliage (i.e., the bacterial aggregates). Each culture tube also contained one complex three-dimensional structure that resembled a parachute.

At higher magnification using the confocal microscope, the thick fibers in the flocculent material appeared tightly coiled. The tightly coiled structures contained bacteria and had an affinity for fluorescently-labeled concanavalin A (conA). These results suggest that there are specialized zones within the biofilm consisting of bacteria associated with extracellular proteins. The presence of bacterial aggregates in the biofilm that did not label with con A suggests that at least part of the extracellular material contains glycoproteins.

Rapid freezing of biofilms followed by freeze substitution and epoxy resin embedding of the specimens enabled examination of thin sections through biofilms that had been minimally disturbed [35,36]. Cryofixation followed by freeze-substitution has been shown to be a highly effective method for preserving biofilm organization for EM examination [37]. It is well known, however, that freezing can lead to structural artifacts [38] and that highly hydrated structures such as biofilms will collapse to some extent during sample preparation that involves dehydration. These distinct features must be recognized to avoid misinterpretation of the images. The fact that complex three-dimensional structures were observed in fully hydrated living specimens using a dissecting microscope, a compound microscope, and with a confocal microscope is strongly suggestive that the biofilm structures observed here are real, and not the result of either sample preparation or specimen handling. The same structures also were present in rapidly frozen, freeze-substituted material that has been embedded in resin.

The results presented in this preliminary account are derived from monospecies biofilms, grown in the laboratory under artificial conditions. Biofilms produced *in situ*, either in the environment or in medical specimens, usually consist of more than one species or subspecies, sometimes making up highly complex microbial communities. The extracellular ultrastructures of such multispecies biofilms could differ from that of the monospecies model biofilms studied here by forming a more heterogeneous matrix, or by providing substrates for catabolic processes in other species. Therefore, it is possible that the observed high degree of matrix organization could be the result of growing pure cultures under constant conditions and may not be as pronounced in the environment. More research on multispecies biofilms observed *in vitro *as well as those taken directly from natural environments is required to thoroughly address this important issue.

The biofilms were characterized in terms of their overall chemical composition (Table 1) and were found to consist primarily (up to 49% wt) of proteins, reflecting the typical dry weight composition of *E. coli *cells under balanced growth conditions [39]. Polysaccharides were found to make up a smaller fraction of the biofilm mass (ca. 15% wt), and were of the magnitude expected in a vegetative bacterial cell. These results are atypical for EPS produced by Pseudomonads, which generally have a higher sugar-protein ratio. *Pseudomonas aeruginosa *is considered a model organism for biofilm research and consequently has been studied intensively within this context [40]. The EPS of *P. aeruginosa *SG81 consists primarily of uronic acids (alignate) and proteins, in roughly a 2:1 ratio (by weight, sugar-protein) [41]. Marcotte *et al*. reported sugar-protein weight ratios of 0.79 for *P. aeruginosa*, where-as the intracellular sugar-protein weight ratios for two *P. aeruginosa *strains were in the 0.27–0.36 range [29]. It should be noted that the biofilms in these studies were processed by different methods to those described here. The comparison of sugar-protein ratios, however, still is relevant and underscores the difference in chemical composition of the biofilms produced by these related Pseudomonads. Alginates in biofilm EPS have been implicated in the development and maintenance of the mechanical stability of biofilms formed by *P. aeruginosa *both on living and abiotic surfaces [42]. The lack of observed *O*- or *N*-acetylation in the biofilm samples analyzed here also is noteworthy, as these groups are common components of biofilm EPS produced by *Pseudomonas *spp. [28].

Total nucleic acid levels in the biofilm (ca. 5% wt) were one order of magnitude higher than corresponding DNA measurements (ca. 0.5% wt). This was not unexpected as total nucleic acid levels will include contributions from RNA; *E. coli *cells typically contain six times more RNA than DNA [39]. The nucleic acid mass fraction of the studied biofilms, however, was ca. 5 times lower than the nucleic acid dry weight content of *E. coli*.

The calcium content (3% wt) of *P. fluorescens *EvS4-B1 biofilm equaled the total dry weight of **all inorganic ions **typically found in *E. coli *[39] and was three times higher than the calcium content of the spent media. Korstens *et al*. studied the mechanical properties of *P. aeruginosa *biofilms as a function of calcium ion concentration and found that the apparent Young's modulus, representing a measure of biofilm stiffness, increased strongly at a critical calcium concentration and subsequently remained constant at higher calcium levels [43]. This behavior was explained in terms of calcium ions crosslinking EPS components. Based on these results it is conceivable that the observed calcium accumulation in the biofilms studied here plays a significant role in crosslinking/bridging EPS components and herewith determining the geometry and maintaining the integrity of the observed structures. Unlike calcium, magnesium was not found to accumulate significantly in the biofilms relative to the spent media.

Note that the chemical composition of the biofilm presented in Table 1 is a semi-quantitative approximation rather than a rigorous, absolute quantitation, which is virtually impossible as the chemical heterogeneity of bacterial biofilms [44] precludes representative standards to be used in a number of the above assays.

Cell and colony morphology have been used by microbiologists in the identification of bacteria since van Leeuwenhoek developed the optical microscope nearly three hundred and fifty years ago. The morphology of bacterial biofilms also may contain elements that can assist identification, but the features can only be observed under the electron microscope. The difficulty in preparing biofilm samples for examination by this technique without introducing artifacts has limited its usefulness. The emergence of cryomethods such as those described here has enabled the reliable application of electron microscopy to biofilm research. Recent results suggest that bacterial biofilms contain architectural motifs that may be useful in identifying these structures in medical, dental, and environmental samples. This approach has been used by Costerton and colleagues in studying intraamniotic infections [45] and affected bone in patients with osteonecrosis of the jaws secondary to bisphosphonate therapy [46]. Biofilms produced by *P. fluorescens *EvS4-B1, *P. putida *[27], and *P. fulva *(data to be presented elsewhere) isolates from the same environment share a common morphology suggesting that these microscopic features may be useful for *in vivo *identification.

## Conclusion

The present work provides evidence that a *Pseudomonas *strain isolated from a natural soil environment can self-organize into elaborate biofilm constructs made up of an extracellular matrix of repeating motifs. Mature biofilms contained living bacteria and were structurally, chemically, and physiologically heterogeneous. These remarkable structures are formed in the laboratory without unusual culturing conditions (i.e., beyond the choice of medium, temperature, and incubating conditions) and the organism does not appear to lose the ability to form biofilm, even after a six or more subcultures. The principal architectural elements observed by electron microscopy may be useful morphological identifiers for classifying bacterial biofilms *in vivo*.

The complexity and reproducibility of the structural motifs in the observed biofilms suggest that they are the result of organized assembly and not a result of *ad hoc *associations. These results suggest possible ecological advantages of the *P. fluorescens *EvS4-B1 strain. Cooperation among microbes currently is generating much interest within both the evolutionary and microbial communities [47]. The matrix of cross-linked polymers observed in the studied biofilms is being produced in copious amounts with high associated costs to the bacteria, while causing large separations between cells. These are relevant and impressive observations, especially within the context of recent theoretical studies [48], which have demonstrated that polymer production in biofilms can be a competitive trait allowing EPS-producing bacteria to occupy more favorable locations in the biofilm while "suffocating" strains of non-polymer producers. Conversely, biofilm EPS may provide a protective microenvironment fostering mutualism, such as encountered among endophytic bacteria that colonize intercellular spaces in various interior plant tissues and in the rhizosphere without causing damage. It has been suggested that biofilms produced by facultative endophytes may be involved in protecting plants from vascular pathogens and may have applications in pesticide phytoremediation [49]. Begun *et al*. showed that EPS from staphylococcal biofilms protected the enclosed bacterial communities against the immune defenses of the model nematode *Caenorhabditis elegans *[50].

## Methods

### Bacterial isolation and culture conditions

The bacteria used in this study were isolated from soil (*T *= 31.6°C) directly adjacent to a tar seep at a location on Sulphur Mountain (Ventura County, CA). The soil isolate EvS4-B1 was obtained following enrichment on solid media containing 10 μM thioanisole using the minimum number of passes required to obtain a pure culture. Working cultures of the EvS4-B1 isolate were maintained as slants on complex media inoculated directly from cryostocks. Slants were discarded two weeks following inoculation.

Strain EvS4-B1 was cultured using a freshwater medium lacking essential vitamins and minerals (10 mL, see below) in 20 mm culture tubes. Cultures were maintained at 30°C and were shaken at 250 rev min^-1^. The same growth medium was used throughout.

Freshwater minimal medium, with essential vitamins and minerals omitted, (FW-Min, NVNM) consists of: Na_2_C_4_H_4_O_4_.6H_2_O (2.7 g.L^-1^), Na_2_SO_3 _(0.14 g.L^-1^), 100X FW base (10 mL.L^-1^), and MOPS (1 M, 5 mL.L^-1^, pH 7.2). 100× FW base consists of: NaCl (100 g.L^-1^), KCl (50 g.L^-1^), MgCl_2_.6H_2_O (40 g.L^-1^), CaCl_2_.2H_2_O (10 g.L^-1^), NH_4_Cl (25 g.L^-1^), and KH_2_PO_4 _(acidic, 20 g.L^-1^). Deionized water (*DI*-H_2_O) was used throughout.

### Identification of the bacterial strain

Genomic DNA was extracted using a rapid desalting process (MasterPure Complete DNA and RNA Purification Kit, Epicentre Biotechnologies, Madison, WI) and samples were prepared following the protocols provided. PCR amplification of the genomic DNA was carried out using two primer types: (1) universal primer pair [51], 63f (CAGGCCTAACACATGCAAGTC) and 1387r (GGGCGGWGTGTACAAGGC) (Invitrogen Life Sciences, Carlsbad, CA); and (2) Pseudomonas-specific primer pair [52], Ps-for (59-GGTCTGAGAGGATGATCAGT-39) and Ps-rev (59-TTAGCTCCACCTCGCGGC-39) (Invitrogen Life Sciences, Carlsbad, CA). The PCR system consisted of 1 μL undiluted template, 1 μL 200 μM dNTP mixture, 1 μL (20 pmol) primer (each), 5 μL buffer (from Taq polymerase kit, see below), 1 μL Taq polymerase (ELT PCR System, Roche Applied Science, Indianapolis, IN). The mixture was diluted to a final volume of 50 μL using MilliQ-H_2_O. Initial denaturation was achieved by heating the mixture at 95°C for 1–2 min, followed by 30 cycles of the following thermal profile: denaturation, 95°C, 30 s; annealing, 57°C, 30 s; and polymerization, 72°C, 60 s.

The PCR product was analyzed by agarose gel electrophoresis (100 V, 20 min) using a 1.2% agarose gel containing ethidium bromide (7 μL in 50 mL of agarose) in a 1× TAE buffer. The most intense band in the gels was cut and purified using a PCR gel extraction kit (QIAquick, QIAGEN Sciences, Germantown, MD). Sequences were determined by the California Institute of Technology Sequencing Analysis Facility using a Model 3730 DNA Analyzer (Applied Biosystems, Foster City, CA) and ABI BigDye terminator cycle sequencing chemistry with the same primer pair as used in the PCR. The partial sequences were analyzed with the Basic Local Alignment Search Tool (BLAST) and compared to BLASTN nucleotide databases [53]. BLAST analysis was used to determine the closest known relatives by comparison with sequences contained in the GenBank database. The purity of the sequence was assessed visually using Chromas 2.3 (Technelysium Pty Ltd, Tewantin, Qld, Australia). The sequence data have been submitted to the GenBank database under accession number FJ226759.

Complementary metabolic tests were carried out with a commercial identification system (API 20 NE, bioMérieux, Inc., Durham, NC) following the manufacturer's instructions. Fatty acid analyses were obtained (MIDI Labs, Inc., Newark, DE) from single bacterial colonies grown on TSA following derivatization as the methyl esters and analysis by GC/MS [54,55].

### Scanning electron microscopy (SEM)

Biofilms (1 to 3 weeks old cultures, depending on the experiment) were removed from culture tubes and prepared for SEM as described previously [36]. The dried biofilms were mounted on metal specimen stubs, coated with a 16 nm thick platinum film, and imaged using an XL-30 S FEG SEM (FEI Company, Hillsboro, OR) operating at 5 kV.

### Transmission electron microscopy (TEM)

Bacterial biofilms (1 to 3 weeks old cultures, depending on the experiment) were immobilized by rapid freezing [56], dehydrated by freeze-substitution in cold acetone containing glutaraldehyde (1% v/v, from a 10% stock solution in acetone; EMS Hatfield, PA) and osmium tetroxide (1% w/v) [57-59] and embedded in resin. Rapid freezing was achieved either by using a high-pressure freezer (EMPACT2 HPF, Leica Microsystems, Inc, Deerfield, IL) or by immersion in liquid propane. Thin sections were prepared from different regions of the embedded specimen blocks, stained with uranyl acetate and lead citrate, and were examined in a TEM (CM 120 BioTwin, FEI, Inc., Hillsboro, OR).

### Biofilm chemical analysis

Supernatant spent media was decanted from biofilms (1 week old culture) at the bottom of the culture tubes. A glass Pasteur pipette was then used to aspirate the complete biofilm from the tube and collected in a 12 mm glass test tube. Biofilms from 17 culture tubes were combined in this fashion. Biofilm-free spent media (5 × 2 mL in 12 mm tubes) and the combined biofilm samples were freeze-dried overnight in a SpeedVac concentrator (SVC100H, Savant, Thermo Fisher Scientific, Inc., Waltham, MA) equipped with a refrigerated condensation trap. SDS-buffer consisting of 1 mM Tris/Tris HCl, 0.1 mM EDTA, 0.15 M NaCl, 1% w/v SDS with a final pH (unadjusted) of 7.51 at 25°C was used to dissolve freeze-dried biofilm/media samples (10 mg in 3 mL) with sonication until a pale yellow solution was obtained.

Dry biofilm and media samples were analyzed for calcium and magnesium content by ICP-AES (Galbraith Laboratories, Inc., Knoxville, TN). IR absorption spectra were collected on an FTIR spectrometer (Magna-IR 560, Nicolet, Madison, WI) as 12 mm diameter discs using ca. 3 mg of dry sample in ca. 150 mg of potassium bromide. UV spectra of the SDS-buffer solutions were obtained using a Model 8452A (Hewlett-Packard, Palo Alto, CA) diode array spectrophotometer in a 1 cm optical path with SDS-buffer as a reference. Total carbohydrate concentrations were measured as previously described [41,60]. These measurements were carried out on suspensions of solid biofilm/media samples in *DI*-H_2_O because SDS-buffer interfered with the assay. Dextrose monohydrate in *DI*-H_2_O (21.3 mg in 100 mL) was used as a stock solution to prepare standards. The absorbances at 480 nm (acidic polysaccharides) and at 490 nm (neutral polysaccharides) were corrected with the absorbance at 600 nm. Protein and nucleic acid concentrations were estimated from the baselined UV spectra [61,62]. Protein concentrations in SDS-buffered samples were measured using a modified Lowry Protein Assay Kit (23240, Pierce, Rockford, IL) according to manufacturer's instructions. Calibration standards were prepared from the supplied BSA standard (2.0 mg mL^-1^) using pipettors and SDS-buffer as the diluent. The DNA content of SDS-buffered samples was estimated according to the method described by Brunk *et al*. [63] using a fluorescence spectrophotometer (F-4500, Hitachi, Schaumburg, IL) with deoxyribonucleic acid sodium salt from salmon testes (D1626, Sigma, Milwaukee, WI, 2.4 mg in 100 mL SDS-buffer) as the standard.

Volumetric concentrations of mixed biofilm/media samples were converted into mass concentration, which were corrected according to eq. 1 for contributions from spent media to afford analyte levels in the biofilms.

where [*y*]^*M*^_*mix *_is the mass concentration of substance *y *in the biofilm/media mixture; [*y*]^*M*^_*biofilm *_is the mass concentration of substance *y *in the biofilm; X_*biofilm *_is the mass fraction of substance *y *in the biofilm; [*y*]^*M*^_*media *_is the mass concentration of substance *y *in the media; X_*media *_is the mass fraction of substance *y *in the media.

### Confocal laser scanning microscopy

Biofilms (1 to 3 weeks old, depending on the experiment) were removed from culture tubes and placed in the depression of concavity microscope slides (EMS, Hadfield, PA). The bacterial material was incubated in the presence of fluorescent dyes, rinsed, covered, and the living, hydrated biofilms were examined by confocal microscopy (SP5 high speed spectral confocal microscope, Leica Microsystems, Inc, Deerfield, IL).

### Image processing and manipulation

All images in this study were digitally captured and manipulated to adjust image size, contrast and brightness. Linear adjustment of size, contrast or brightness was always applied equally to the entire image.

## Authors' contributions

AK, TO'K, and RP participated in all aspects of the reported laboratory studies with a special emphasis on bacterial isolation, cultivation, and genetic sequencing. KM participated in the design and analysis of results from the rapid freezing experiments. SW participated in the microscopy laboratory work. MMB and PW conceived of the study, and participated in its design and coordination. All authors read and approved the final manuscript.

## Supplementary Material

Additional file 1 **Additional material for: characterization of structures in biofilms formed by *Pseudomonas fluorescens *isolated from soil**. The data provided includes a fourteen-day growth curve for *P. fluorescens *EvS4-B1 and peak assignment for the FTIR absorption spectra of dry media/biofilm samples. Click here for file

Additional file 2 **EvS4-B1 Grown in minimal media**. Movie of mature *P. fluorescens *EvS4-B1 biofilms in a 10 mL culture tube. Click here for file
