# Supplementary material for: Characterization of structures in biofilms formed by a Pseudomonas fluorescens isolated from soil
Source: BMC Microbiol. 2009 May 21;9:103. doi: 10.1186/1471-2180-9-103 (PMC2697165; doi:10.1186/1471-2180-9-103)
Supplement: Additional file 1 — Additional material for: characterization of structures in biofilms formed by Pseudomonas fluorescens isolated from soil. The data provided includes a fourteen-day growth curve for P. fluorescens EvS4-B1 and peak assignment for the FTIR absorption spectra of dry media/biofilm samples. [file 1471-2180-9-103-S1.pdf]

Additional Material for:

Characterization of structures in biofilms formed by *Pseudomonas fluorescens* isolated from soil

Marc M. Baum[1]*, Aleksandra Kainović[1], Teresa O'Keeffe[1], Ragini Pandita[1], Kent McDonald[2], Siva Wu[3], and Paul Webster[3]

[1]Department of Chemistry, Oak Crest Institute of Science, 2275 E. Foothill Blvd., Pasadena, CA 91107, United States of America,

[2]Electron Microscope Lab, 26 Giannini Hall, University of California, Berkeley, Berkeley, CA 94720, United States of America

[3]House Ear Research Institute, 2100 W. 3rd Street, Los Angeles, CA 90057, United States of America

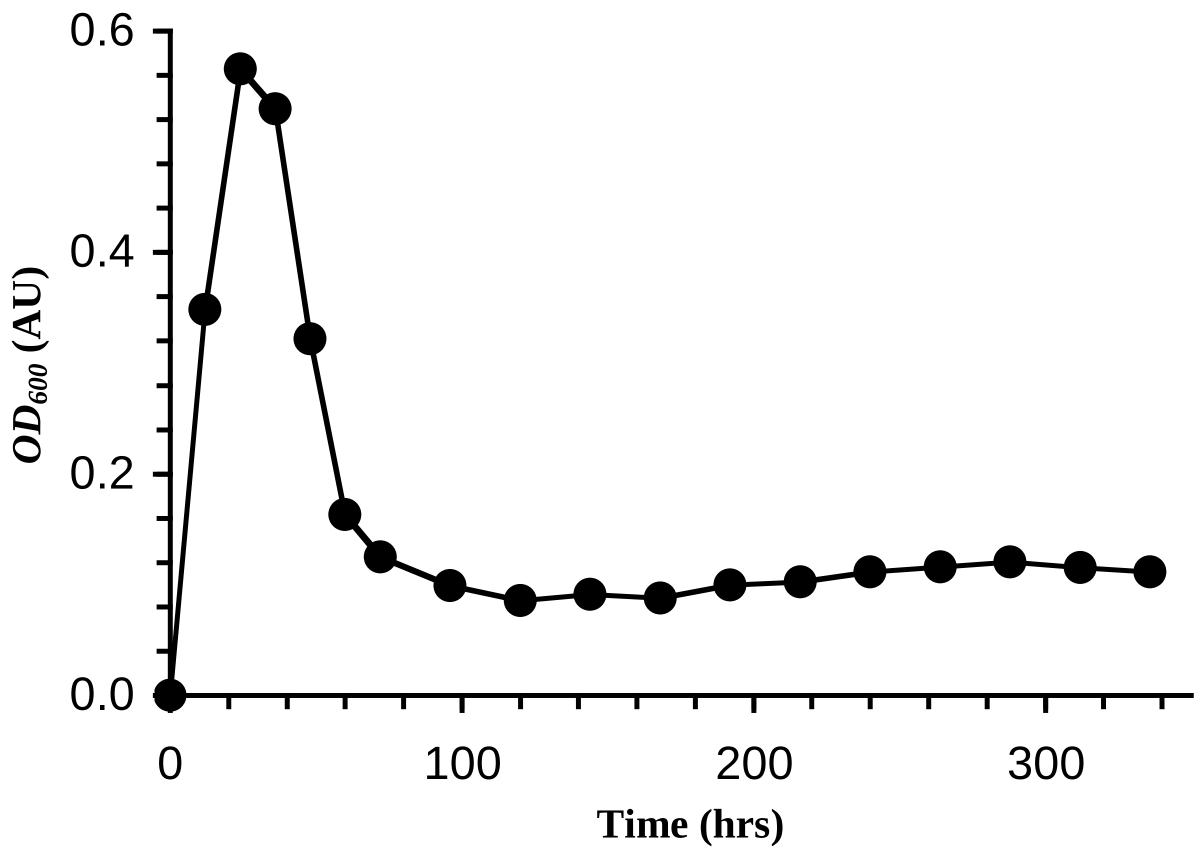

Figure AM-1 - Fourteen-day growth curve (*OD_{600}* represents corrected absorbance at 600 nm; mean of six samples cultivated in parallel) of *P. fluorescens* EvS4-B1 cultivated at 30 °C and 250 rev.min^{-1}.

The absolute values of OD_{600}, and the standard deviation across the six samples, are highly dependent on the characteristics (e.g., age and method of preparation) of the inoculum.

Table AM-1 - FTIR Absorption spectrum of dry biofilm/media sample as a suspension in KBr.

Peak (cm^{-1})	Height[a] (AU)	Assignment
3326, sh	0.171	O-H stretch [1]
2961	0.025	C-H stretch [1]
2930	0.027	C-H stretch [1]
1651	0.182	Amide I, 1661-1665 cm^{-1} [2]; 1650 cm^{-1} [3]
1536	0.045	Amide II, 1547 cm^{-1} [2]; 1530-1560 cm^{-1} [3]; 1480-1590 cm^{-1} [4]
1451	0.020	C-H bending of CH_2 [4]
1400	0.029	CH_3 sym deformation [5]; sym CO_2^- stretch in amino acids [5]
1315	0.003	C-O stretch [1]; *β-D*-mannuronate in alginate [6]
1275, sh	0.001	C-O stretch [1]; *α-L*-guluronate in alginate [6]
1261	0.006	C-O stretch [1]
1239	0.010	Acetate ester C-O stretch [5]; P=O stretch [5]
1220	0.010	Acetate ester C-O stretch [5]; P=O stretch [5]
1170	0.002	C-C and C-O stretch [1, 2]
1113	0.013	C-OH stretch and C-O-C C-O ring vibrations 1100 cm^{-1} in carbohydrates [4]
1045	0.100	C-OH & P-O stretches [7]

[a]Baselined.

br, broad; sh, shoulder.

References

1. Sartori C, Finch DS, Ralph B, Gilding K: **Determination of the Cation Content of Alginate Thin Films by FTIR Spectroscopy**. *Polymer* 1997, **38**(1):43-51.
2. Beech I, Hanjagsit L, Kalaji M, Neal AL, Zinkevich V: **Chemical and Structural Characterization of Exopolymers Produced by *Pseudomonas sp.* NCIMB 2021 in Continuous Culture**. *Microbiology-UK* 1999, **145**:1491-1497.
3. Marcotte L, Kegelaer G, Sandt C, Barbeau J, Lafleur M: **An Alternative Infrared Spectroscopy Assay for the Quantification of Polysaccharides in Bacterial Samples**. *Anal Biochem* 2007, **361**(1):7-14.
4. Serra D, Bosch A, Russo DM, Rodriguez ME, Zorreguieta A, Schmitt J, Naumann D, Yantorno O: **Continuous Nondestructive Monitoring of *Bordetella pertussis* Biofilms by Fourier Transform Infrared Spectroscopy and other Corroborative Techniques**. *Anal Bioanal Chem* 2007, **387**(5):1759-1767.
5. Lin-Vien D, Colthup NB, Fateley WG, Grasselli JG: **The Handbook of Infrared and Raman Characteristic Frequencies of Organic Molecules**. San Diego: Academic Press; 1991.
6. Filippov MP, Kohn R: **Determination of Composition of Alginates by Infrared Spectroscopic Method**. *Chem Zvesti* 1974, **28**(6):817-819.
7. Karadenizli A, Kolayli F, Ergen K: **A Novel Application of Fourier-transformed Infrared Spectroscopy: Classification of Slime from *Staphylococci***. *Biofouling* 2007, **23**(1):63-71.

Additional Multimedia

Video clip (B1_MinMedia.wmv) shows biofilm material (*P. fluorescens* EvS4-B1, shaking culture) *in situ*.
